# Spread of domestic animals across Neolithic western Anatolia: New zooarchaeological evidence from Uğurlu Höyük, the island of Gökçeada, Turkey

**DOI:** 10.1371/journal.pone.0186519

**Published:** 2017-10-18

**Authors:** Levent Atici, Suzanne E. Pilaar Birch, Burçin Erdoğu

**Affiliations:** 1 Department of Anthropology, University of Nevada, Las Vegas, Las Vegas, Nevada, United States of America; 2 Department of Anthropology & Department of Geography, University of Georgia, Athens, Georgia, United States of America; 3 Department of Archaeology, University of Thrace, Edirne, Turkey; University at Buffalo - The State University of New York, UNITED STATES

## Abstract

The zooarchaeological research presented here investigates Neolithic and Chalcolithic (ca. 6500–5000 cal. BC) animal exploitation strategies at Uğurlu Höyük on the Turkish island of Gökçeada in the northeastern Aegean Sea. Toward this end, we first discuss the results of our analysis of the zooarchaeological assemblages from Uğurlu Höyük and then consider the data within a wider regional explanatory framework using a diachronic approach, comparing them with those from western and northwestern Anatolian sites. The first settlers of Gökçeada were farmers who introduced domestic sheep, goats, cattle and pigs to the island as early as 6500 years BC. Our results align well with recently published zooarchaeological data on the westward spread of domestic animals across Turkey and the Neolithization of southeast Europe. Using an island site as a case study, we independently confirm that the dispersal of early farming was a polynucleated and multidirectional phenomenon that did not sweep across the land, replace everything on its way, and deliver the same “Neolithic package” everywhere. Instead, this complex process generated a diversity of human-animal interactions. Thus, studying the dispersal of early farmers from southwest Asia into southeast Europe via Anatolia requires a rigorous methodological approach to develop a fine-resolution picture of the variability seen in human adaptations and dispersals within complex and rapidly changing environmental and cultural settings. For this, the whole spectrum of human-animal interactions must be fully documented for each sub-region of southwest Asia and the circum-Mediterranean.

## Introduction

The revolutionary economic and social transformation of societies from foraging to farming in Southwest Asia shortly after 10,000 calibrated years BC (BC hereafter) and the subsequent spread of new genes, languages, ideologies, and domesticated cereals and livestock into Europe via a process called *Neolithization* from 10,000–7000 BC have been the subjects of extensive scholarly debate since the 1970s (e.g., [1, 2]). Various models have drawn on multiple lines of converging evidence including genetics, linguistics, and archaeology to explain the global dispersal of early farming populations with fully developed agropastoral lifeways from primary to secondary centers of agricultural origin (e.g.[3, 4–14]).

Uğurlu Höyük is a Neolithic settlement on Gökçeada (Imbros in Greek), the largest Turkish island situated between Anatolia and the European continent in the Aegean Sea, and currently the only site with an early Neolithic component in the eastern Aegean. Thus, with its key geographical location between Southeast Europe and Southwest Asia and its early Neolithic strata, the results of zooarchaeological research presented here, more broadly, may have implications reaching beyond Anatolia and contribute to our understanding of the spread and development of agricultural societies in southeast Europe in general and the eastern Aegean in particular.

More specifically, this paper focuses on animal exploitation strategies at Uğurlu and adds new zooarchaeological data to the existing body of research on the spread of domesticated animals across Neolithic western Anatolia. We address the following specific questions:

1. Did the islanders have a diverse subsistence strategy, including foraging and marine resource exploitation, or did they heavily rely on livestock management? How did the animal economy change through time?
2. How did island habitation affect animal management decisions compared to the mainland Anatolia? Did the islanders manage cattle, sheep, goats, and pigs differently?

In investigating animal exploitation at the site, we first characterize faunal assemblages and examine their formation processes and taphonomic histories, which assist us in identifying the role of humans in the accumulation, modification, and destruction of these assemblages. Second, we examine taxonomic composition and species trends, body part distributions, age structures, and body size to probe the foregoing questions.

## Conceptual framework and theoretical background

In studying the dispersal of agricultural economies from southwest Asia to southeast Europe, archaeologists have used a dichotomized framework. The colonization or demic diffusion model entails replacement of foragers by advancing waves of farmers [15–17], whereas the indigenous adoption or cultural diffusion model argues for a process of acculturation instead of endemic population movement and replacement ([10]and references therein). The colonization or demic diffusion model hinges on the basis of the materialistic similarity with Anatolia, the general absence of Mesolithic occupation on the eastern Mediterranean islands, and clear genetic presence of the descendants of Near Eastern colonists in extant European populations (e.g. [15–17, 18, 19]). The proponents of the latter model place emphasis on the explicit evidence for pre-pottery Neolithic with Mesolithic affinities ([19]and references therein).

There has been a recent movement, however, toward a consensus acknowledging the complexity of the processes and mechanisms that spread the Neolithic across Europe. Toward this end, it is now recognized that farming spread into Europe by a mixture of expansion, diffusion, and adoption as the predominant mechanisms [20–25]. Neolithic dispersal was not a unidirectional phenomenon sweeping across the land, replacing everything on its way, and delivering the “Neolithic package” of domesticated plants and animals, ground stone tools and ceramics everywhere simultaneously with the same components. Özdoğan [9, 26], Souvatzi [25], and Perlès [23] concur that different regions in southeast Europe followed different rates of adoption of agriculture and sedentism and that multiple Neolithic packages successively spread from central and northwestern Anatolia to Europe. On the other hand, some researchers suggest that the term Neolithic package is misleading and does not reflect the heterogeneity and variety of the Neolithic lifeways [27–29].

## Neolithic subsistence economy in western Anatolia

Benjamin Arbuckle and Levent Atici’s [30] broad survey of demographic and osteometric data combining 78 faunal assemblages from the southwest Asian Neolithic sites dating to around tenth through eight millennia BC documents a high degree of diversity in early caprine management. More specifically, Arbuckle and Atici identify that the ubiquity in the intensive culling of young males, a marker for the initiation of sheep and goat herding, was established and widely practiced across southwest Asia only after 7500 BC ([30]: 232).

In order to expand the corpus of demographic and osteometric data from Anatolia and to document the movement of sheep, goat, cattle, and pig from southwest Asia into Europe through western Anatolia, eighteen researchers working in Turkey merged their primary datasets into a single database representing seventeen sites, 42 chronological phases, and more than 200,000 faunal specimens [6].

In central Anatolia, domestic sheep and goats first appeared by the mid-eighth millennium BC, then domestic cattle a millennium later; domestic pigs were never incorporated into Neolithic economies in this region [31]. Outside of central Anatolia, all four livestock species appeared for the first time in the Izmir region around 6800 BC (Ulucak Level IV), suggesting a rapid westward movement of domestic animals following a coastal route [6]. The fact that Neolithic communities in northwest Anatolia around 6600 BC utilized caprines and cattle but did not keep domestic pigs, whereas communities in the Lake District of southwest Anatolia around 6500 BC utilized all four livestock species, further complicates the matter [32, 33]. The absence of pigs in northwest Anatolia suggests central Anatolian influences, whereas the Lake District and western Anatolia, with all four livestock species, display a developmental trajectory independent from central Anatolia. These multiple lines of evidence suggest that the movement of animal economies involved a complex, multidirectional process.

Furthermore, recent Neolithic investigations in western Turkey have dramatically changed our understanding of the emergence and development of animal husbandry in western Turkey. Excavations at Ulucak Höyük have revealed Aceramic Neolithic layers and pushed the appearance of herding back to the early seventh millennium BC [34, 35]. Sedentary herders, who heavily relied on sheep, goats, cattle, and pigs, with no ceramic tradition and scarce evidence for hunting, appear in the Izmir region around 6800 BC [32]. The earliest evidence for intensive management of sheep, goats, cattle, and pigs together with wild goat and boar hunting and ceramic tradition appears in the Lake District around 7000 BC, creating a contrasting scenario [36, 37]. In the Marmara region, a tripartite herding strategy focusing on domestic sheep, goats, and cattle can be detected in the zooarchaeological record around 6600 BC; the exploitation of suids remains marginal until pig rearing is adopted around ca. 6000 BC [33]. Although the reasons behind the distinct choices in the adoption of pigs remain debatable, palaeogenetic research shows that early European domestic pigs had their origins in western Turkey [37]. For the most part, the earliest Neolithic on the Aegean islands tends to be dominated by imported domesticates—primarily sheep and goat—rather than endemic fauna [38].

The results of biomarker and zooarchaeological research in western Turkey synergize to offer new insights into the relative importance of animal products such as meat and dairy during the Neolithic. The analysis of lipid residues in archaeological ceramics identified dairy use in the Marmara and Izmir regions beginning around 6500 BC [39]. In the Marmara region, a dairy production-oriented cattle rearing is inferred from the large cattle NISP counts [40]. Mortality profiles of domesticated ruminants at Ulucak also suggest dairy production starting around 6500 BC, confirming the results of lipid residue analysis, with little evidence for differentiation in sheep and goat management strategies [38, 41, 42].

**Fig 1:**
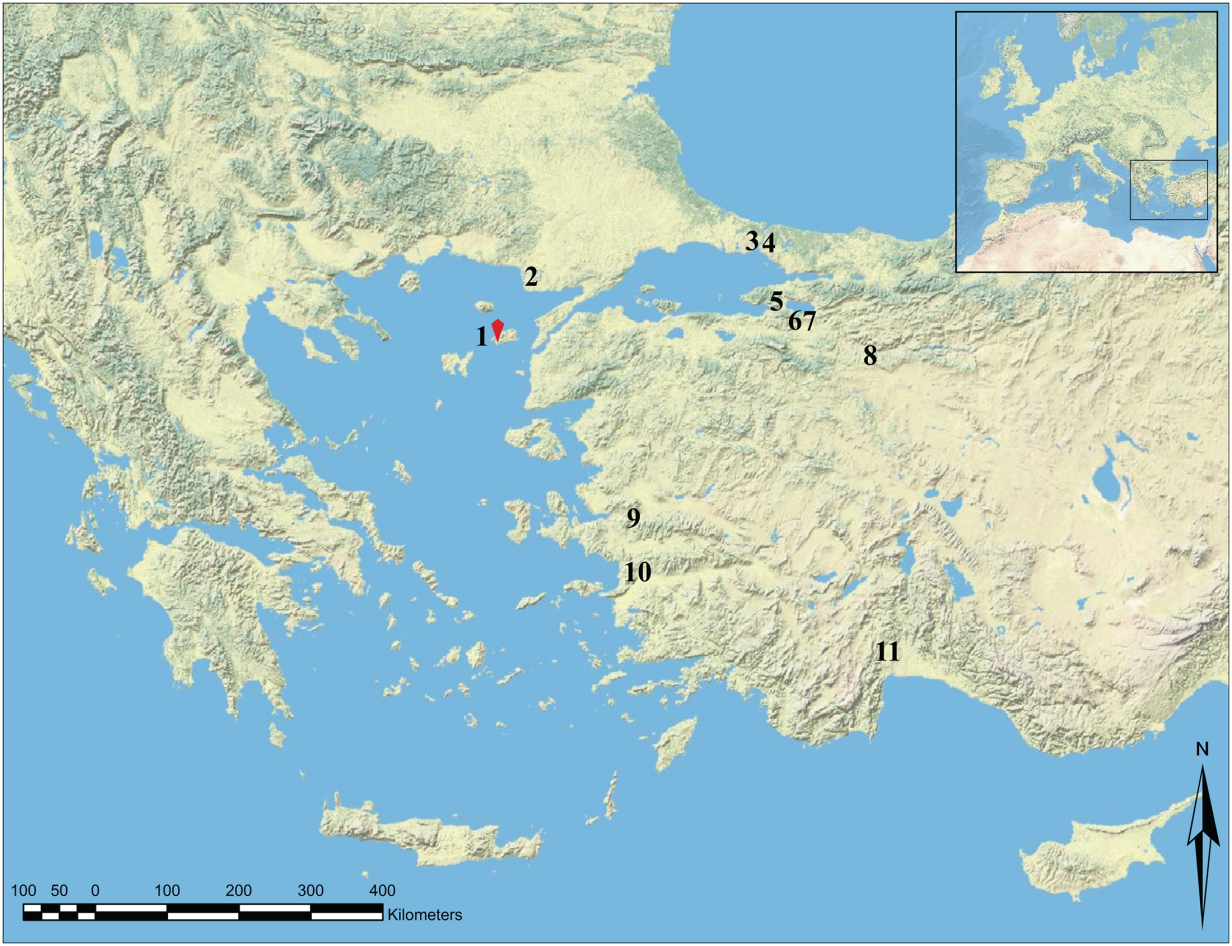
Location of the sites mentioned in the text: 1 = Uğurlu Höyük, 2 = Hoca Çeşme, 3 = Yeni Kapı, 4 = Fikirtepe, 5 = Ilıpınar, 6 = Menteşe Höyük, 7 = Barçın Höyük, 8 = Orman Fidanlığı, 9 = Ulucak Höyük, 10 = Çukuriçi Höyük, and 11 = Karain B and Öküzini caves.

This paper employs an analytical approach similar to that of Arbuckle and colleagues [6] in an attempt to (1) add a new site to the ‘big data’ corpus, (2) extend the scope of that database spatially to go beyond the mainland Anatolia, and (3) include an island settlement to compare and contrast animal exploitation strategies between the mainland Anatolia and the island of Gökçeada. Toward these goals, this paper compares the results of zooarchaeological analyses at Uğurlu Höyük with those from western and northwestern Anatolian sites such as Ulucak Höyük, Menteşe Höyük, Çukuriçi Höyük, Ilıpınar, Barçın Höyük, Fikirtepe, and Hoca Çeşme (See Fig 1 for site locations).

## Site description

The island of Gökçeada lies about 17 km from the Gelibolu (Gallipoli) Peninsula of the Anatolian mainland, and covers an area of 289.5 square km. During the Last Glacial Maximum (ca. 20.000–18.000 BC), sea levels were about 120 m lower than the present sea level [43, 44]. Gökçeada, together with Lemnos, Bozcaada (Tenedos), and Samothrace, was connected to the mainland. During this period, the straits of Dardanelles and Bosphorus were blocked by land and the Black Sea and the Sea of Marmara were no more than fresh water lakes with considerably lower levels than today. Between 12,500 and 10,000 BC, Lemnos and Gökçeada would have become separated from the mainland as sea level rose, though the islands were connected to each other by an isthmus [45, 46]. During the early Neolithic period around 6500 BC, the island of Gökçeada was probably much closer to the mainland when the first settlers sailed there.

The site of Uğurlu Höyük is a low mound covering an area of approximately 250 x 200 m on a gentle slope at the eastern foot of Mount Isa (Doğanlı) on the western part of the island. The main Uğurlu-Dereköy road cuts through the site and it has also been damaged by a long trench dug for the opening of an irrigation system. The Pilon stream lies at the eastern part of the site, and there is a nearby spring. The island is mountainous, and the bedrock geology is mainly composed of volcanic rocks. The site was first discovered in 1998 and a long-term project was started in the summer of 2009 by Burçin Erdoğu [47]. During the six excavation seasons, six main cultural phases, designated as I-VI from top to bottom, and at least 12 layers of occupation have been revealed [48]. The earliest three phases (VI-IV) date to the Neolithic period. Phase III is marked by the Neolithic-Chalcolithic transition, while the succeeding Phase II dates to Chalcolithic and has revealed at least two occupational layers of Western Anatolian Kumtepe Ia-Beşik Sivritepe Culture. Scattered sherds from the Early Bronze Age and Medieval times have been found on the surface, Phase I.

## Chronology, excavations, and findings

Thanks to a rigorous dating program, we have a well-dated and established chronology for the cultural sequence (S1 Table). The earliest stratum Phase VI is dated to between 6700 and 6500, Phase V between 6500 and 6000, Phase IV between 5900 and 5500, and Phase III between 5400 and 4900 BC.

**Fig 2:**
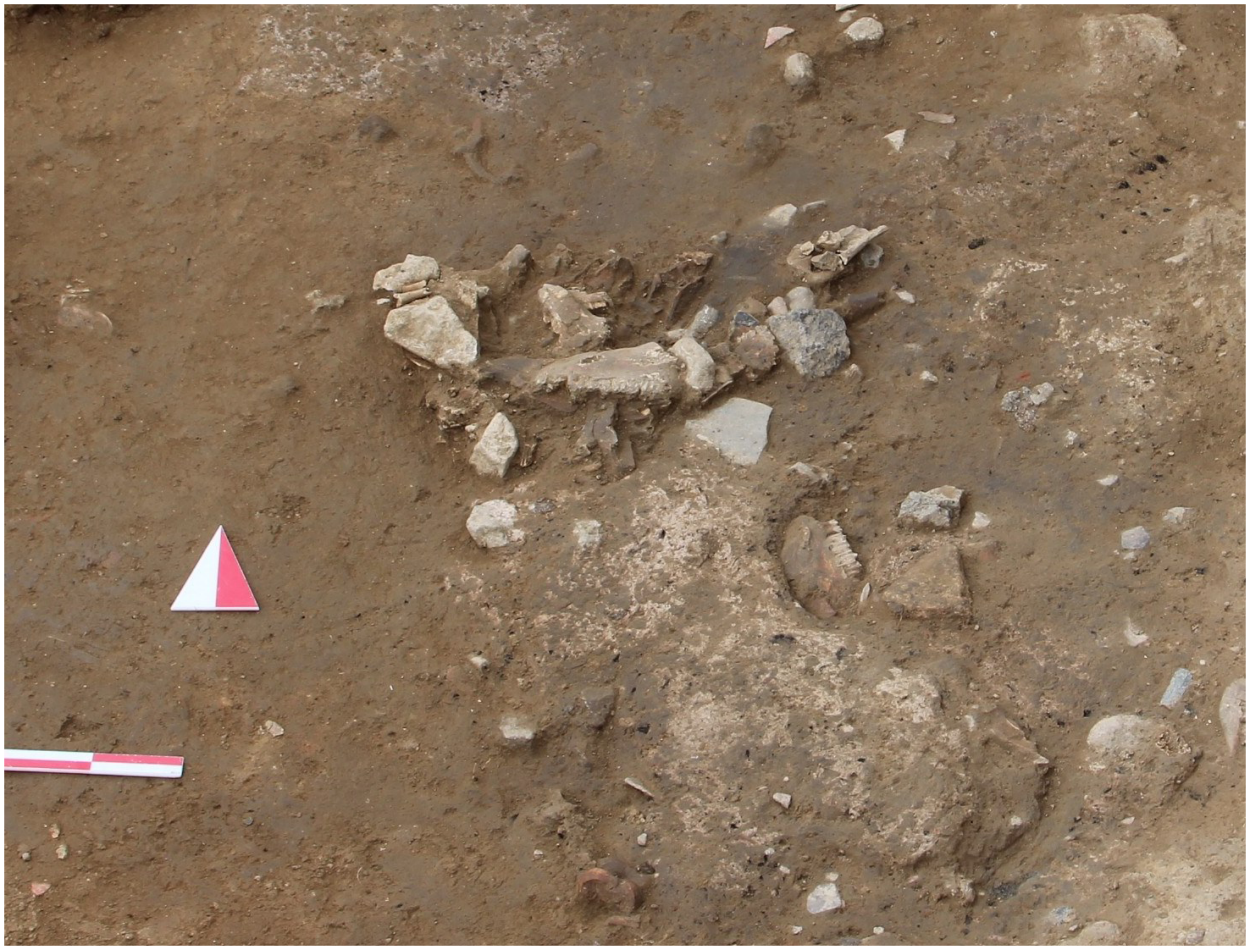
An *in situ* bone cluster from the Phase V at Uğurlu Höyük.

The earliest occupation at the site, Phase VI, is represented in sounding trenches, yielding scattered stones, a hearth, several disc-shaped shell or stone beads and bone awls [48]. This study does not include faunal assemblages from this phase. Phase V occupation is located near the stream on the eastern part of the settlement. To date, two possible occupational layers of Phase V have been identified. The first and earlier layer is represented in deep sounding trenches and characterized by scattered stone clusters and extremely dense bone concentrations (Fig 2). No architectural remains were recorded here. The second and later layer of Phase V has yielded a building with a single-room about 5 x 4 m in size with earthen floor. The building features thick, cobbled walls, a massive exterior buttress, a courtyard, and a one-meter-diameter oven in an open area. The architecture indicates a small-scale household, limited space for social interaction, and no dedicated storage installations.

During Phase IV the settlement seems to have enlarged, covering an area of 6 hectares. Phase IV is represented by a 2.5-meter thick deposit with at least four occupational layers. No complete buildings have been exposed. So far, two layers of Phase IV have been excavated. The first layer has yielded a compact floor, a circular hearth, extremely dense concentrations of animal bones, and bone tools such as awls, chisels, and needles. A building with damaged stone walls, a thick compact floor with multiple layers of plaster and post-holes, a large storage vessel, and an antler hammer were found in the second layer. In addition, mats preserved as phytoliths were sitting on some parts of the floor.

**Fig 3:**
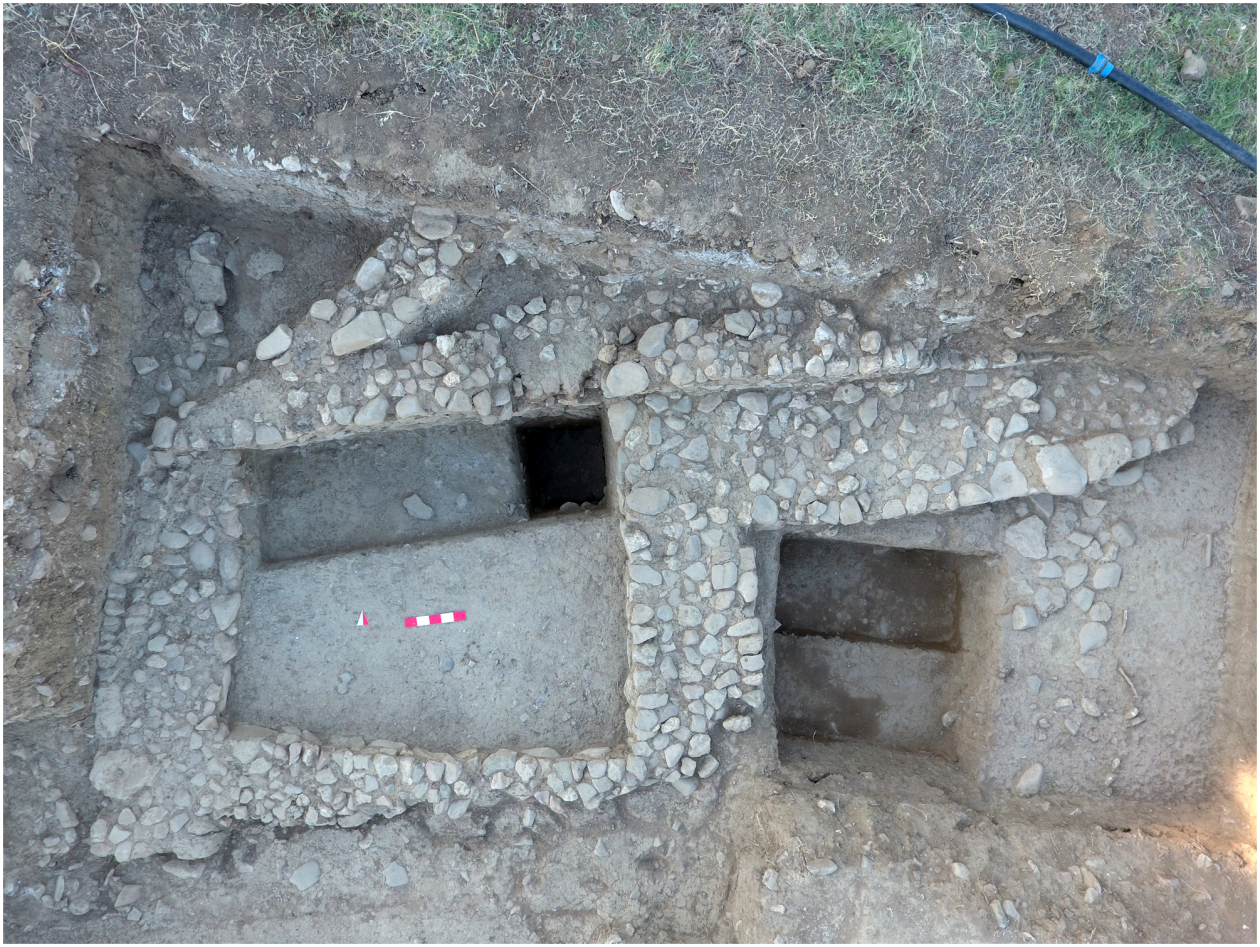
Architectural remains from the Phase IV at Uğurlu Höyük.

In the eastern part of the site, a building with a long exterior buttress and two architectural phases has been partly excavated (Fig 3). From the late phase, a floor with shell beads, polished stone axes and adzes, bone awls, smoothers, a bone hook, and a marble mace head have been unearthed. A sounding trench has yielded a courtyard with at least 5 hearths and carbonized botanical remains of domesticated cereals including Einkorn wheat (*Triticum monococcum*), six-rowed barley (*Hordeum vulgare)*, *naked barley* (*Hordeum vulgare* var. *nudum*), and pea (*Pisum sativum L*.). Large quantities of shells and some fish bones indicate the potential role of marine resources in the Neolithic diet of Uğurlu. Among shells, *Patella* and Mytilidae are numerous. Neolithic figurines make up a small assemblage that includes acrolithic figurines, a marble figurine head, and a pregnant-like anthropomorphic figurine.

**Fig 4:**
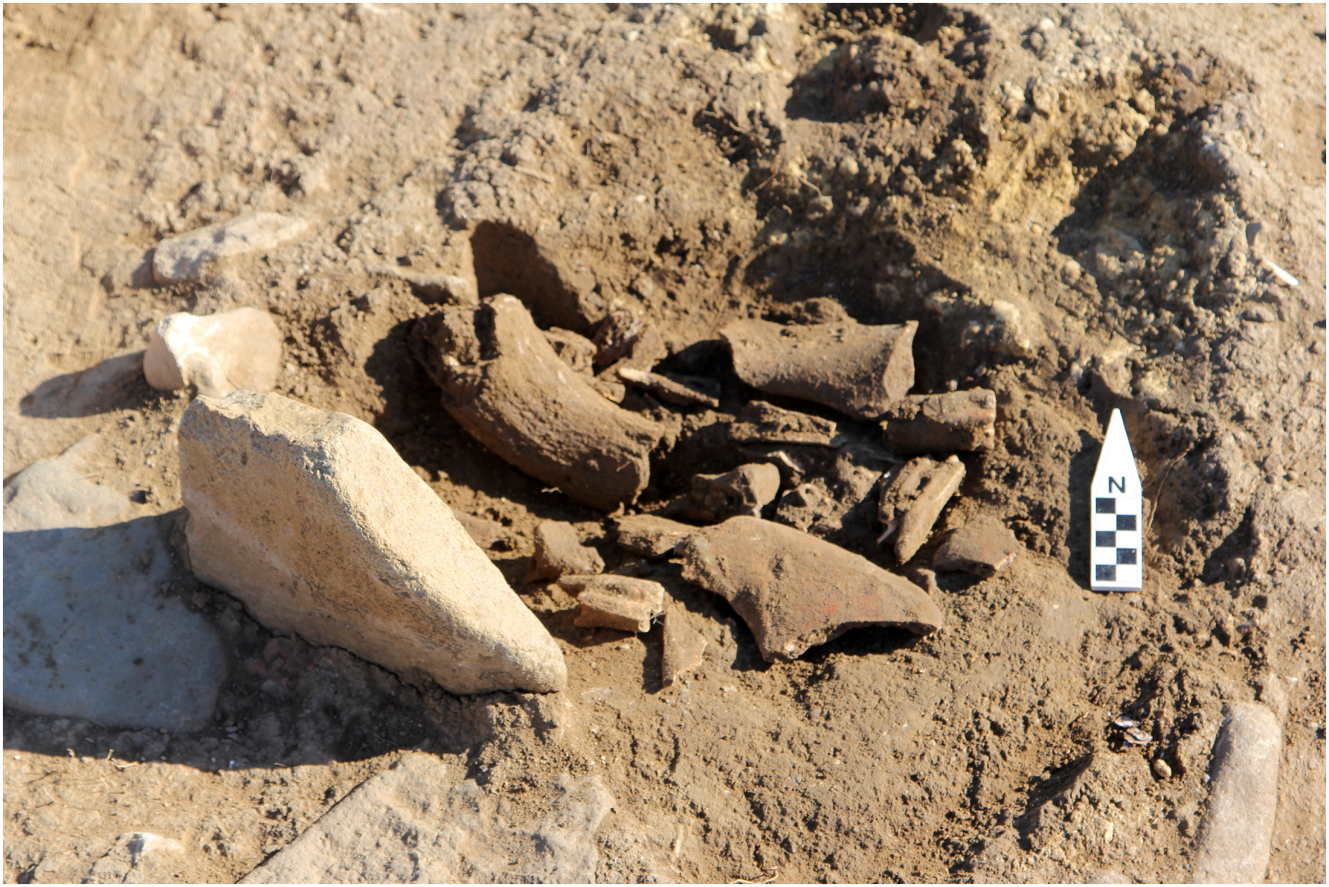
*In situ* animal remains from the Phase III at Uğurlu Höyük.

Phase III is represented by a rectangular building with red painted lime plaster floor and a bull horn on its entrance and 25 plastered pits that are circular in shape with a depth and diameter of 1 meter. The pits were deliberately refilled with large stones before abandonment and yielded animal bones, pottery sherds, bracelets or rings made from *Spondylus gaederopys*, pendants made from *Cerastoderma*, and bone and antler tools (Fig 4). In addition, a secondary burial of a middle-aged male bearing the traces of red ochre pigments was found. Phase III has also yielded large number of anthropomorphic figurines [49].

One distinctive aspect of Neolithic Uğurlu was long-distance exchange, best reflected in the distribution of obsidian and Balkan flint. The most distinctive tool type in phases IV and III is a flint macro blade, the so-called “Karanovo macro blade” [50]. About 25 macro blades were found in this phase and a core and some flakes from the so-called Balkan flint were also recovered. Obsidians were analyzed by Marina Milic using portable X-Ray Fluorescence. The results demonstrate that the obsidian comes from 3 sources: one the island of Melos, and the two others East Göllü Dağ and Nenezi Dağ of Central Anatolia. While Melian samples are much more frequent, an obsidian bullet core from the Nenezi source is unique.

## Zooarchaeological methodology

Permission to carry out the archaeological fieldwork that yielded the datasets used in this project was provided by the Turkish Ministry of Culture and Tourism. All the zooarchaeological specimens involved are under the auspices of the Turkish Ministry of Culture and Tourism and are permanently stored in the Uğurlu Höyük Excavation Project Dig House on the island.

### Recovery and sampling

Despite the lack of systematic dry- or wet-screening, all the excavated sediments were scrutinized to ensure full recovery of macro and microfaunal remains and to minimize the biases involved in the recovery of the assemblage. Faunal assemblages from a total of 20 archaeological contexts representing strata V, IV, and III (9, 7, and 4 contexts, respectively) were sampled randomly, generating 6061 bone fragments. Of the three strata, Phase V has generated the largest sample (N = 3967), as the faunal remains were densely packed in a small area of 2 x 4 m, enabling effective hand-picking. This area lies at the edge of the residential areas of the settlement, and might have served as a disposal area for the Neolithic inhabitants. The sediment in this area is almost totally built of bones, making up 90 to 95% of the matrix, with sporadic lithic and pottery elements. The size distribution of shaft fragments smaller than 5 cm in each of the three strata, with 85, 86, and 73%, respectively, attests to the overall efficiency of recovery at the site.

### Recording

The recording protocol employed in this work entailed general documentation of the entire assemblage for the purpose of characterization and included every element, element portion, and nonidentified splinter recovered (N = 6061). No pre-sorting was practiced and all of the bones were packed and stored together in the storage area of the Uğurlu Höyük dig house. Every fragment was examined first by naked eye and then with a 10–15 x hand lens under strong light, if necessary, for bone surface modifications, while sub-samples were randomly chosen for recording variables such as fracture platform angle and percussion and notches. All the fragments were identified to the maximum degree possible, refitted and mended when possible, weighed, counted, labeled, assigned unique individual specimen numbers, measured when appropriate, and entered into an automated FileMaker database [51]. When individual recording of fragments was not necessary, grouped specimens were counted, weighed, and entered into the database as a single entry under the same specimen number (e.g., nonidentified long bone shaft fragments, nonidentified skull fragments, and splinters). Recording took place at the project’s facilities near the site on the island during field seasons 2011, 2013, and 2014 by Levent Atici, and in 2015 by Levent Atici and Suzanne Pilaar Birch.

### Identification

Taxonomic and skeletal element identifications were carried out partly using a modern comparative reference collection assembled by the authors and partly using published manuals and articles describing identification criteria. When the degree of certainty of identification was high, specimens were identified to the highest taxonomic category possible, i.e., species. When identification to a higher taxonomic category such as species, genus, or family was not possible, methodological categories, such as “medium artiodactyl” were used. For the purpose of statistical viability, the bones of sheep and goats were combined into an “O/C” (“caprine”) category and treated as a single analytical unit. According to Shipman ([52]: 106), the microscopic bone structures and size of animals determine how their bones break. As such, combining the bones of medium-sized bovids such as sheep and goats for taphonomic purposes should not impact the validity of the taphonomic analysis and results presented here. For mortality curves and size index analyses, however, only the bones identified to species were used.

### Quantification

Number of Fragments (NF), Number of Identified Specimens (NISP), Minimum Number of Elements (MNE), and Bone Weight (BW) were quantitative measures employed in this paper [53]. NF was used to document entire assemblages including non-specific skeletal part categories such as nonidentified bone splinters and long bone shaft fragments, and NISP was used when fragments could be identified to skeletal element and at least to a taxonomic or size category [53, 54]. For the estimation of MNE, a combination of discrete features or landmarks [55] and manual overlap approach [56] were used. Degree of completeness for all the specimens were recorded to achieve a certain degree of standardization and to avoid double-counting and inflating the element numbers. Among other quantitative measures used were the average bone weight for all fragments and the average specimen size for long bone shaft fragments. A recent experimental study has confirmed that these measurements can shed light on the degree of fragmentation [57]. Bone Weight (BW) was used together with NF and MNE to further assess the contribution of taxa to the diet based on the allometric relationship between bone weight and total body weight of an organism ([53]: 94).

In order to probe animal management strategies, we first present the demography of the mortality data based on the state of long bone epiphyseal fusion and dental eruption and wear for caprines. The long bone data (MNE counts) were recorded to document age structures following the fusion sequence and corresponding age brackets that Zeder [58] documented for modern wild caprine (*Ovis orientalis* and *Capra aegagrus*) specimens from Iran. For dental remains, age classes were recorded for caprines following procedures described by Deniz and Payne [59], and the three-cohort system [60] was used due to the high degree of fragmentation and the subsequent absence of large series of mandibles.

Following the age data, we present osteometric or biometric data from Uğurlu Höyük following the standards (i.e., [61]). We compare data from multiple Neolithic sites from western Anatolia using primary datasets or raw measurements, directly taken from the open access, peer reviewed data publishing system Open Context (http://opencontext.org), and/or the Logarithmic Size Index (LSI) values following Richard Meadow [62]. All the datasets used in this paper have citable DOIs/persistent identifiers that are listed in the appropriate supporting data tables and cited accordingly in the bibliography [63–69]. At the most fundamental level, the LSI method entails the comparison of depth and breadth measurements even in the absence of large samples from a single skeletal element and portion in a single archaeological context [62]. This is accomplished by comparing every osteometric observation to the standard comparative reference animal. We used measurements of a modern wild female mouflon (*Ovis orientalis*) from Iran for sheep [70]; average measurements of male and female wild goats (*Capra aegagrus*) from Taurus mountains in Turkey for goats [70]; and measurements of a modern female wild boar from eastern Turkey for pigs [71].

## Results

### Assemblage formation

S2 Table presents the general characteristics of the assemblages. The first step of the analysis reveals the taphonomic history. Bone surface modification analysis systematically included scrutiny of all skeletal parts for traces of carnivore gnawing, acid corrosion, and marks left by rodents, weathering, and root etching. The analysis of 6061 fragments weighing about 26 kg suggests that faunal assemblages from the three strata were all accumulated, modified, and destroyed largely by cultural processes.

A detailed analysis of bone surface modifications has revealed that rodent marks, weathering, and traces of root etching are extremely rare, indicating rapid burial events and intensive occupation and maintenance activities at the site. Direct and indirect traces of carnivore ravaging are almost absent from the Neolithic strata (V and IV), while the Neolithic-Chalcolithic transition phase (III) shows slightly increased carnivore activity at the site. The ratio of cylinders without articular ends, which usually indicates carnivore modification and destruction of nutrient rich and spongier long limb bone articular ends, is also low in the assemblages. The marginal number (N = 5) of red fox bones from Phase V and a single dog bone from Phase III independently support the lack of carnivore involvement in the assemblage formation processes and can partially help account for the lack of their impact as a taphonomic agent. The lack of carnivore impact, in turn, indicates human processing as the primary taphonomic filter.

Not-identified long bone shaft fragments comprise a high percentage, showing a clear bias against mechanically less resistant or less dense elements in general and the axial skeletal elements and long bone articular ends in particular. NF to MNE (NF/MNE) ratios, average fragment size and average fragment weight data combine to suggest that the three assemblages were exhaustively fragmented by humans. The presence of cut marks on almost every skeletal element provides the most direct evidence for human modification of bones and reflects every stage of carcass processing and management from slaughtering to skinning to dismemberment to tendon removal as well as other traces of consumption including marrow extraction, tongue removal, and filleting. The fracture angle data coupled with percussion marks and notches suggest that most bone breakage was the result of dynamic loading or hammer-stone blows when the bones were in a fresh state. Acute and obtuse angles in the sampled shaft assemblages with very high proportions indicate deliberate breakage of bones for marrow extraction.

### Taxonomic composition and species trends

S3 Table elaborates taxonomic composition and relative abundance of taxa based on NF, MNE, and BW counts. The Uğurlu Höyük assemblages reveal that the Neolithic and Chalcolithic inhabitants of the island exploited a wide range of taxa in varying proportions. The remains of bovids dominate the entire cultural sequence, whereas specimens representing suids, cervids, leporids, carnivores, and avifauna are present in varying and insignificant proportions and are not ubiquitous. Hunted or wild taxa include large-bodied (red deer, fallow deer, and wild boar) and small game (European hare). Most of the game animals identified at Uğurlu Höyük come from the Neolithic strata, with level V yielding a majority of this subset. The wild cat, great bustard, and mackerel shark are each represented by a single specimen from stratum IV, whereas a duck/goose specimen from stratum V and a dog specimen from stratum III account for other one-of-a- kind ecofacts from Uğurlu Höyük.

**Fig 5:**
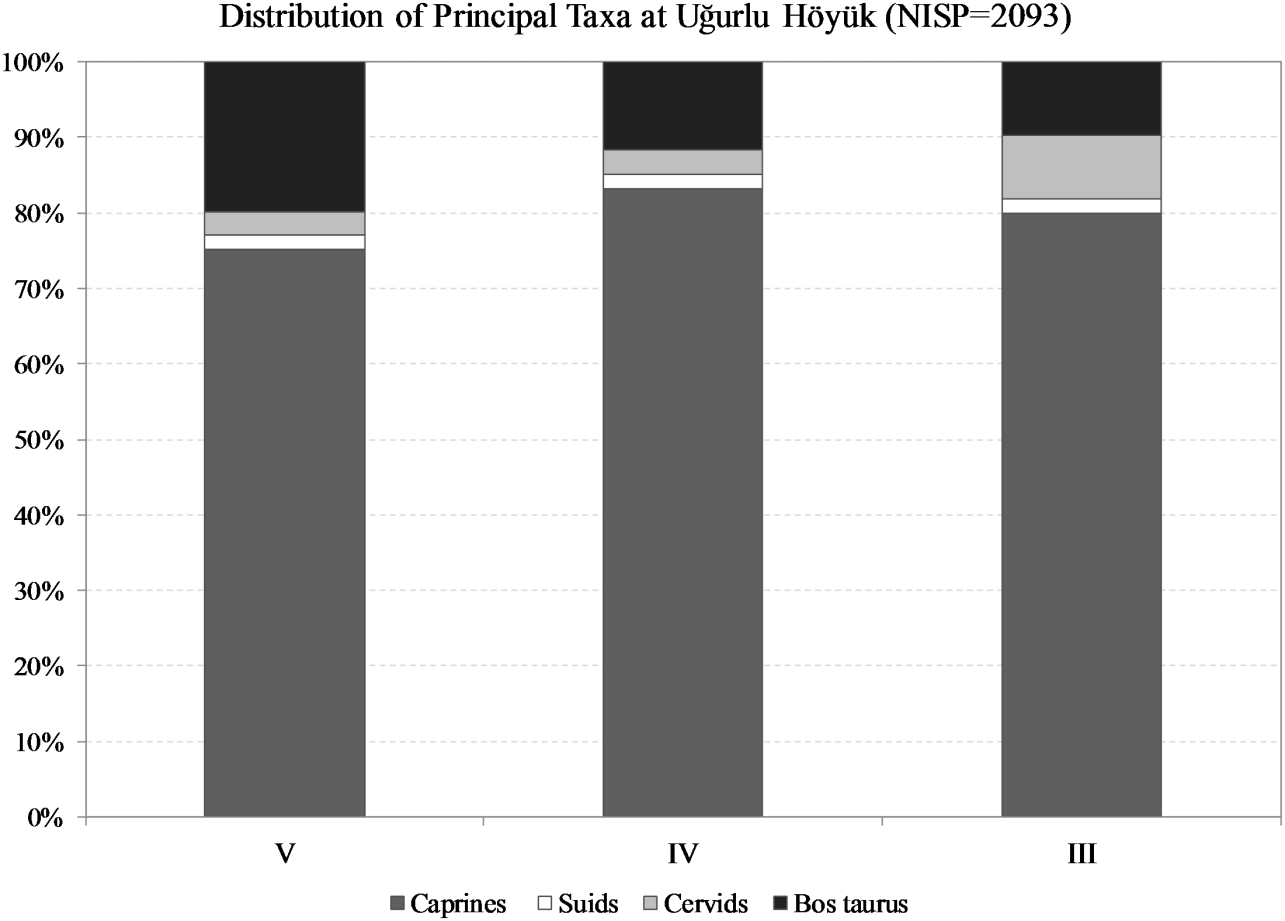
Ratio distribution of principal taxa at Uğurlu Höyük using NISP counts.

The faunal assemblages from Uğurlu are dominated by three principal food animals—sheep, goats, and cattle—as their bones comprise ca. 95% of the Neolithic and 90% of the Neolithic-Chalcolithic strata (Fig 5). Among the three livestock species, caprines seem to be the primary focus of pastoral economy when NF and MNE counts are taken into account, as they are represented in a much higher proportion (varying from 75 to 83% of all the identified bones) than cattle are (varying from 10 to 20% of all the identified bones). When the bone weight data presented in S3 Table are taken into account, however, the patterning changes in favor of cattle, which provide the largest dietary contributions varying from 30 to 53%. Sheep outnumber goats throughout the sequence, although the latter progressively increase from 6% in stratum V to 22% in stratum III, whereas the exploitation of sheep and cattle visibly decline.

**Fig 6:**
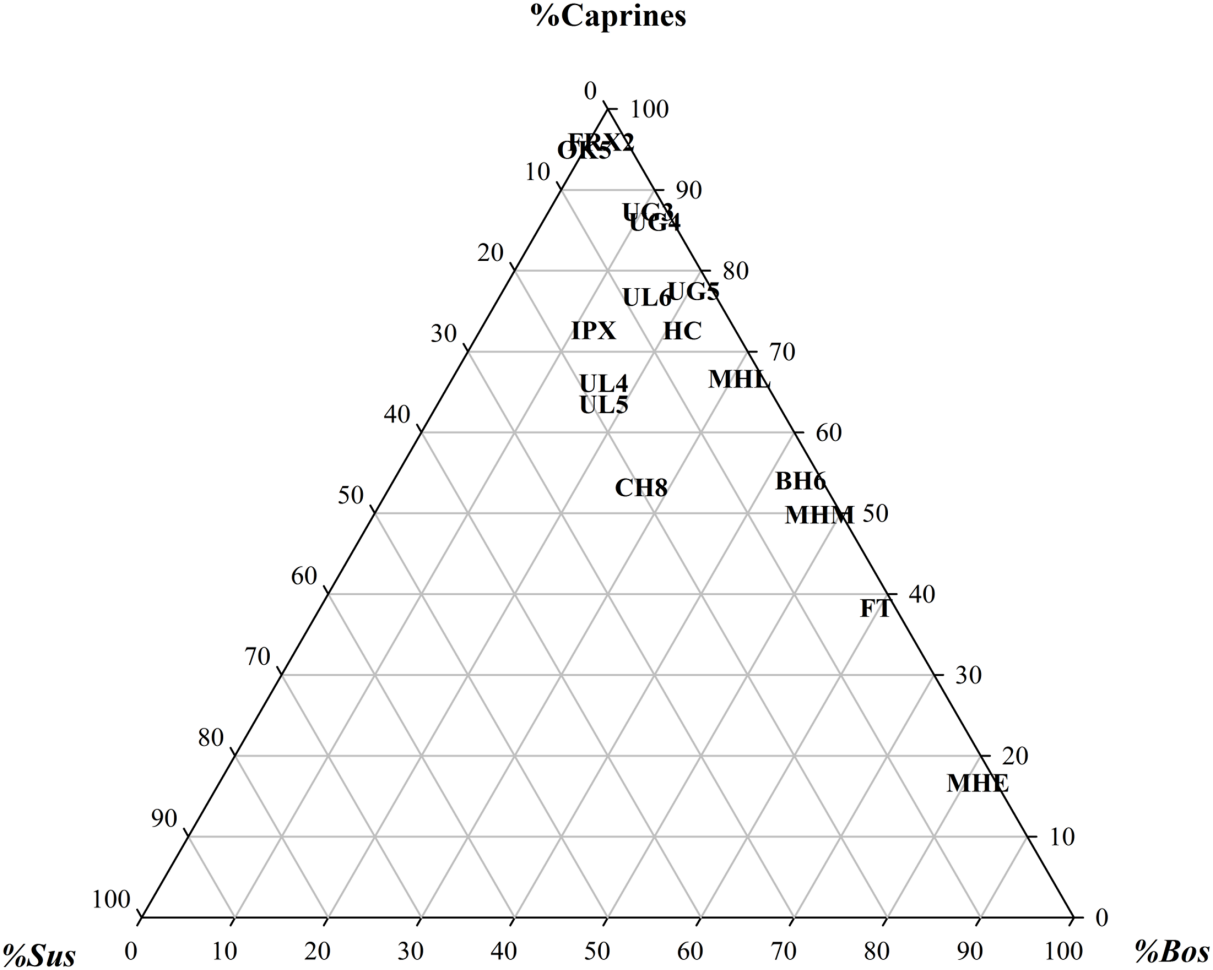
Ternary graph showing ratio distribution of principal taxa in western Anatolia faunal assemblages. Assemblages represented are as follows: OK5 = Öküzini Cave V; UG 5, 4, 3 = Uğurlu Höyük V, IV, III, respectively; UL 6, 5, 4 = Ulucak Höyük VI, V, IV, respectively; IPX = Ilıpınar X; HC = Hoca Çeşme; MHE = Menteşe Höyük Early; MHM = Menteşe Höyük Middle; MHL = Menteşe Höyük Late; BH6 = Barçın Höyük VI; CH8 = Çukuriçi Höyük VIII; FT = Fikirtepe.

In the following section, we place Uğurlu Höyük assemblages within a wider regional framework using a diachronic approach to probe whether spatiotemporal patterning emerges. S4 Table shows the NISP values for caprines (sheep and goats), cattle, and pigs identified in a number of Neolithic sites in Anatolia’s western and northwestern regions, as well as Gökçeada in the northeastern Aegean. The western region is represented by two sites from the Aegean, Ulucak Höyük and Çukuriçi Höyük. The northwestern region comprises two sub-regions: the Marmara and Turkish Thrace, which are represented by assemblages from Barçın Höyük, Fikirtepe, Ilıpınar, and Menteşe Höyük in the former and Hoca Çeşme in the latter. Since we presently do not have Late Pleistocene sites with well-dated sequences or faunal assemblages in the western and northwestern regions of Turkey, Epipaleolithic Karain B and Öküzini caves in the western Taurus Mountains (southwest region) with seven assemblages are included as a baseline to gauge changes in animal exploitation strategies from the late Pleistocene into the early Holocene in Anatolia. The assemblages from the two southwest sites are exclusively dominated by wild caprines, whereas aurochs are completely absent and wild boars are marginally represented (< 1%) except for Öküzini V, the only assemblage plotted here for the sake of making the ternary graph more intelligible [72, 73]. NISP ratio values for the three taxonomic categories were plotted as a tripolar graph to examine whether there is a conspicuous bias against any of the categories (Fig 6). The three vertices of the triangle represent x, y, and z axes, which in turn indicate the proportional relationships among the three taxa.

**Fig 7:**
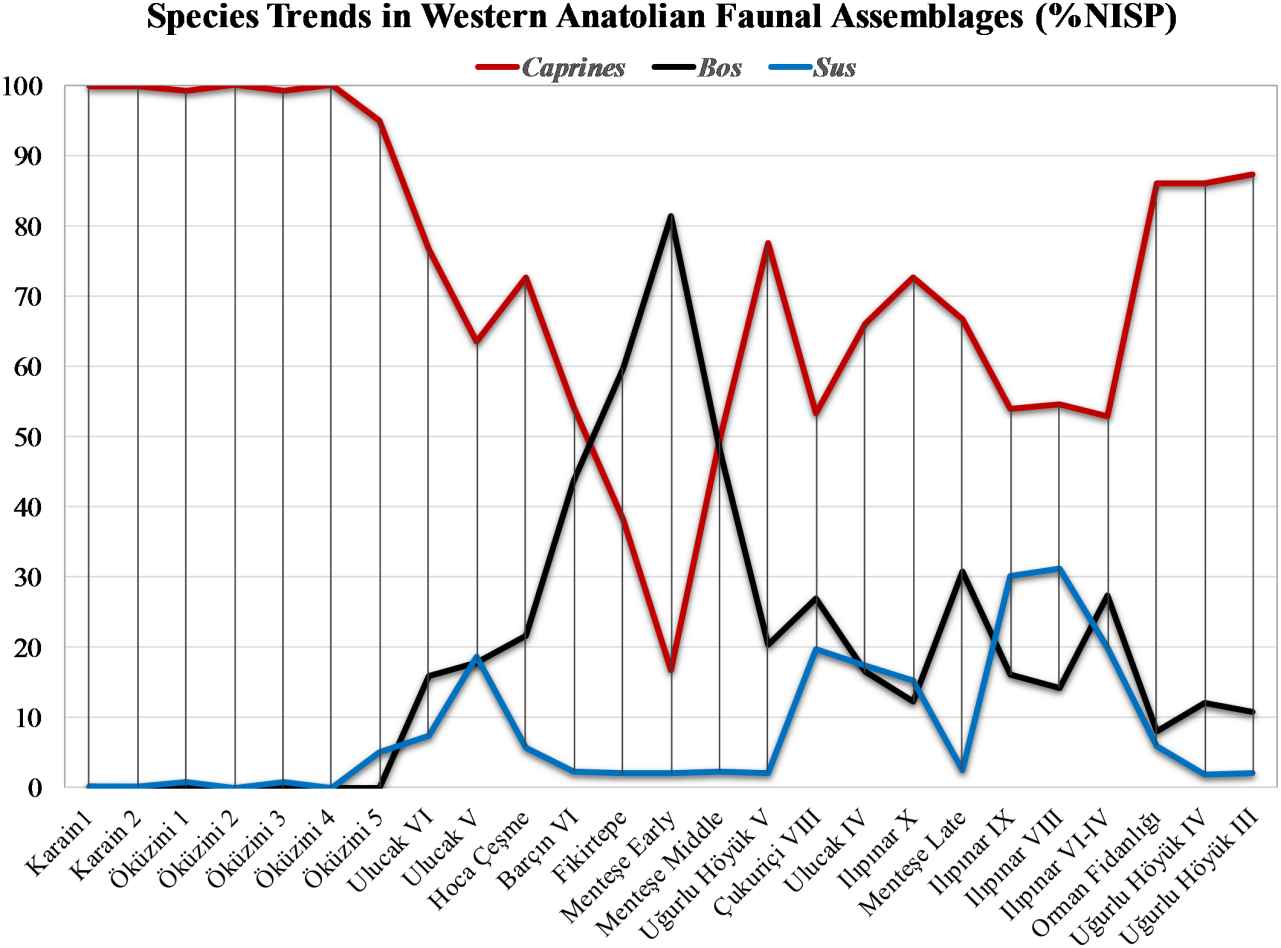
Species trends in western Anatolian faunal assemblages (%NISP).

Ulucak VI, with strata dating to 7000–6500 BC range, represents the earliest Neolithic in the northern Aegean region. As Figs 6 and 7 show, Ulucak VI has a relatively even taxonomic composition compared to Öküzini V, with cattle represented by ca. 16% and pigs at about 7%, which indicates a multitaxic yet monodominant assemblage (sensu [74]). Thus, the earliest phase of Ulucak Höyük is also characterized by a specialized, caprine-focused pastoral economy.

Figs 6 and 7 demonstrate a trajectory in the Aegean region toward progressively increasing taxonomic evenness during the 6500–6000 BC range. At Ulucak V, while there is a slight increase in the proportion of cattle from about 16 to 18%, the sharp increase in the proportion of pigs from about 7 to 19% is notable and at the expense of a similarly notable drop in caprine representation. Slightly later in date, Çukuriçi VIII, too, confirms the departure from a caprine-dominated pastoral economy in the Aegean region. Here, the remains of cattle and pigs account for about 47% (27 and 20%, respectively) of the three-tiered animal economy. When we move to the northwestern region, the three Marmara sites, Fikirtepe, Barçın Höyük, and Menteşe Höyük mirror this trajectory towards increased evenness in the taxonomic composition. Here, too, the departure from heavy reliance on caprine management is evident. But unlike the Western Anatolian region, the focus in the Marmara region shifts to cattle, not to pigs, whose representation drops back to 2%. As such, the differences between western and northwestern regions are significant in scale and divergent in nature. The fact that remains of cattle at Menteşe Höyük Early Phase make up 81% of the entire assemblage is remarkable. Although not as high as proportions observed at Early Menteşe Höyük, Barçın Höyük, Fikirtepe, and Middle Menteşe Höyük assemblages have similar species trends with very high cattle representation ranging from about 44 to 60%. At these sites, too, the most interesting aspect of the overall trend is the significant underrepresentation of pigs. When we move to the Turkish Thrace to examine Hoca Çeşme, we see that species trends here depart from the cattle-dominant Marmara model, as the proportion of cattle drops to about 22%.

Phase V of Uğurlu Höyük shows a pattern that is congruent with Hoca Çeşme in that cattle represent about 20% of the assemblage. Both of these sites also depart from the pattern observed in western region; that is, their apparent lack of an emphasis on pigs. All these combined, an argument for the presence of regional trends can be made based on the taxonomic composition and species trends data.

During the latest phase of Neolithic dating to around 6000–5500 BC, we have assemblages from Ilıpınar, Menteşe Höyük, and Orman Fidanlığı representing the northwestern region, one assemblage from Ulucak Höyük representing the western region, and one assemblage from Uğurlu Höyük representing Gökçeada. Species trends at Ulucak IV shows a conspicuous continuity, mimicking the taxonomic ratios seen in the two earlier phases; primary caprine exploitation (66%) supplemented by secondary cattle (17%) and pig (17%) husbandry. The sites in the Marmara Region show greater taxonomic diversity when compared to the succeeding phase of the Neolithic. At Late Menteşe Höyük, the decline of cattle management continues with a sharp drop from about 48 to 31% while the proportion of caprines climbs from about 50 to 67%. At Ilıpınar, a very clear and visible trend is documented; proportions of caprines gradually decline from about 73% in level X to 55% in level VIII, spanning the end of the Neolithic from 6000 to 5600 BC. This change is marked by a shift from caprines to pigs whose proportions rose from 15 to 31% during the same period (Fig 7).

The final phase, dating to the late Neolithic-early Chalcolithic (ca. 5500–4900 BC) in the northwest region and Gökçeada assemblages, can be said to reflect the same patterning with an increasing diversity in taxonomic configuration. Ilıpınar VI-IV show that the decline in the dominance of caprines continued while a greater evenness between cattle and pigs were established (about 53, 27, and 20%, respectively). Orman Fidanlığı in the northwestern region and Uğurlu Höyük III differ from other northwestern sites with a more distinctively caprine-dependent (86 and 87%, respectively) management system. It seems that animal management at Orman Fidanlığı and Uğurlu Höyük became increasingly more specialized during the initial Chalcolithic (Fig 7).

### Animal exploitation: Carcass management, demography of mortality, and body size

S5 Table shows that all main caprine and cattle body parts are present in the assemblages in varying proportions except for the total absence of axial elements for both taxa in stratum III. This could be a product of small sample size and/or density-mediated attrition targeting less dense axial elements, but even so, this does not indicate any clear patterning, nor does it suggest selective removal, transport or processing of carcasses to primarily focus on more nutritious and meaty skeletal elements. Thus, the analysis of body part distributions indicates that full caprine carcasses were accessed, processed, and consumed. However, small sample sizes and disparities among MNE counts do not permit meaningful body part ratio comparisons between caprines and cattle, pigs, wild boars, fallow deer, and red deer (S5 Table).

With this caveat in mind, the frequency distribution of game contrasts with that of domesticates. Stratum V, with the highest NF (3,967) and MNE (954) counts among the three strata, may provide the most representative picture of body part distribution for game taxa. Here, the elements of forelimb and hind limb comprise 71% of all boar bones, 73% of all fallow deer bones, and 50% of all red deer bones, while the elements of cranial and axial skeletons are either completely absent or significantly underrepresented. Though a smaller sample, Stratum IV, too, mimics the same pattern with the forelimb and hind limb elements comprising 100% of all boar bones, 100% of all hare bones, and the forelimb elements making up 80% of all red deer bones.

**Fig 8:**
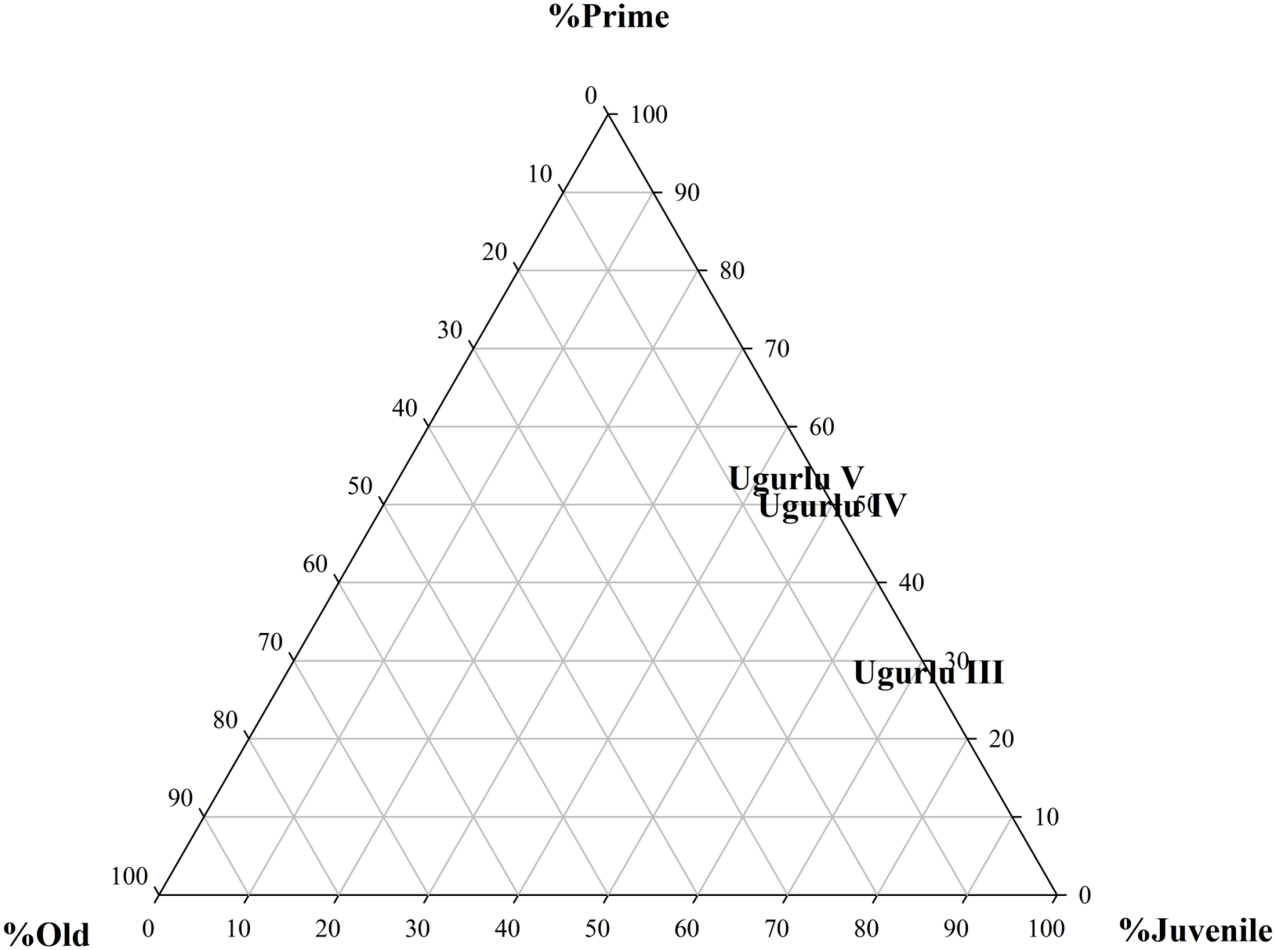
Ternary graph showing caprine demographic trends at Uğurlu Höyük using three-cohort system.

The kill-off patterns or survivorship curves for caprines were generated based both on the age at death estimations of the long bone articular ends and tooth wear data for dp4-P4 or dP4- M3 pairs. Unfortunately, the sample size-related disparities prevented us from generating age-at-death data for pigs, wild boars, fallow deer, and red deer. The epiphyseal fusion data for the earliest Neolithic phase suggest that all the caprine individuals survived the first two months, 77% of the animals survived 6 months of age, 67% survived the first year, 67% survived 18 months of age, and only 37% survived beyond 30 months of age. The pattern reflects an ethnographically documented meat-oriented animal management strategy in which animals are slaughtered between 9 months and 30 months before a potential decline in their meat yield. The data for phases IV and III show the so-called “resurrection” of individuals as a result of sample size-related biases, not permitting meaningful observations and interpretations. The dental wear data plotted on the ternary graph (Fig 8) indicate a progressively younger kill-off pattern from Phase V through III. This, in turn, may be reflecting an increased young male slaughter which is the hallmark of more intensive management of caprines that came to represent specialized pastoral economies of the Near East in later periods [30].

For cattle, the small sample size (N = 87) imposed a cut-off point and permitted the assignment of cattle long bone epiphyseal specimens into either younger or older than 24 month age categories. The analysis of available epiphyseal fusion data for the small sample indicates that less than 30% of cattle survived beyond two years of age during stratum V with an upsurge in age at death to 70% and 50% during the succeeding strata IV and III, respectively. This may be due to the changing role of cattle in subsistence economy and a shift from a primary to secondary animal product-oriented pastoral economy with the institutionalization and intensification of farming during the late Neolithic and early Chalcolithic.

**Fig 9:**
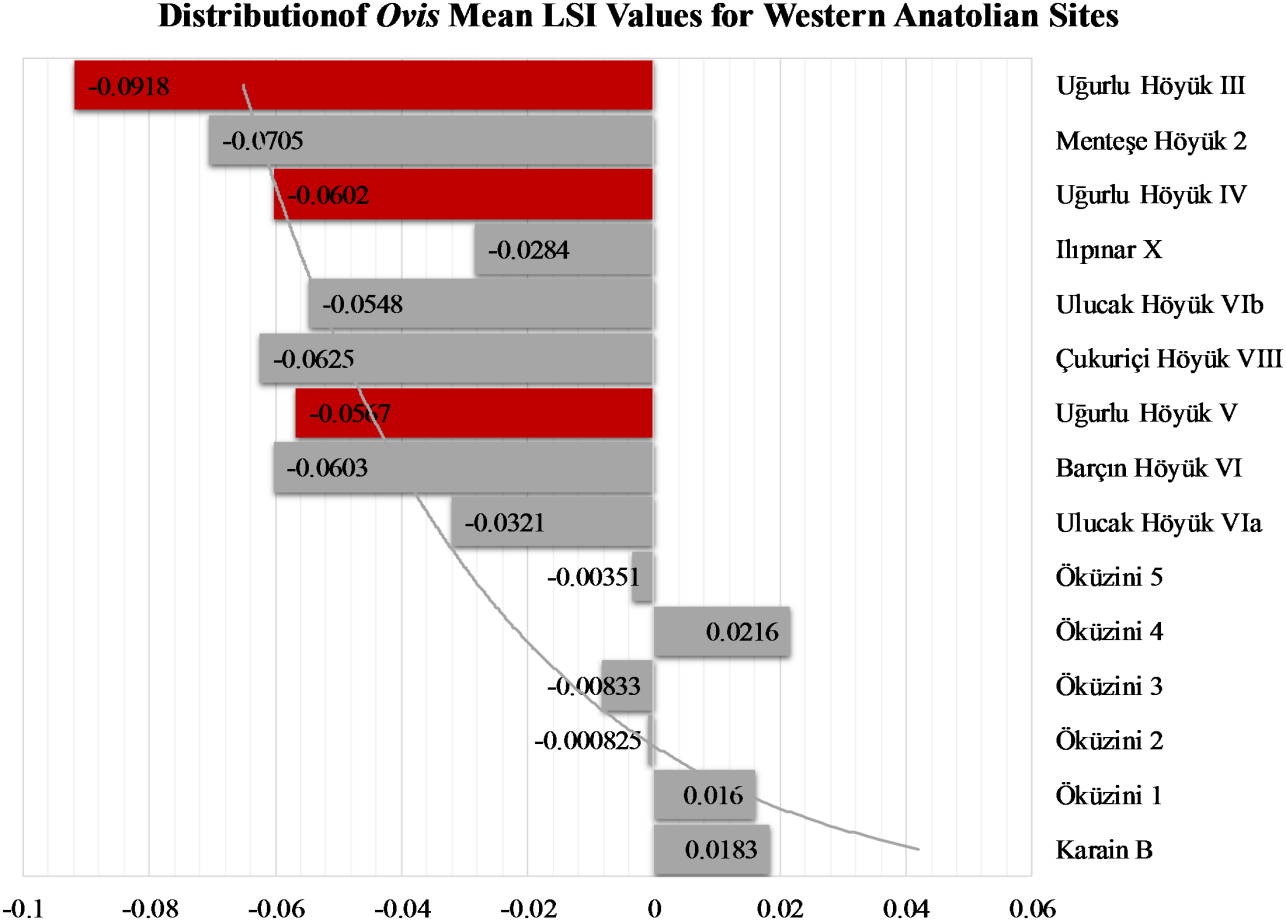
Distribution of *Ovis* mean LSI values for western Anatolian sites.

Although mean sheep LSI values from different Anatolian sub-regions vary conspicuously, the island populations from Gökçeada during the earlier two phases, V and IV, seem to align well with those from Barçın Höyük VI, Çukuriçi Höyük VIII, and Ulucak Höyük VIb (Fig 9; S6 Table). When placed into a longer and wider spatiotemporal framework, it becomes even clearer that Uğurlu Höyük sheep represent one of the more intensively managed domestic phenotypes during the Neolithic. In contrast, sheep populations during the ensuing transitional Chalcolithic phase, III, must have gone through a selective process locally on the island that led to further size reduction to the extent that they sit at the lowest end of the size distribution.

**Fig 10:**
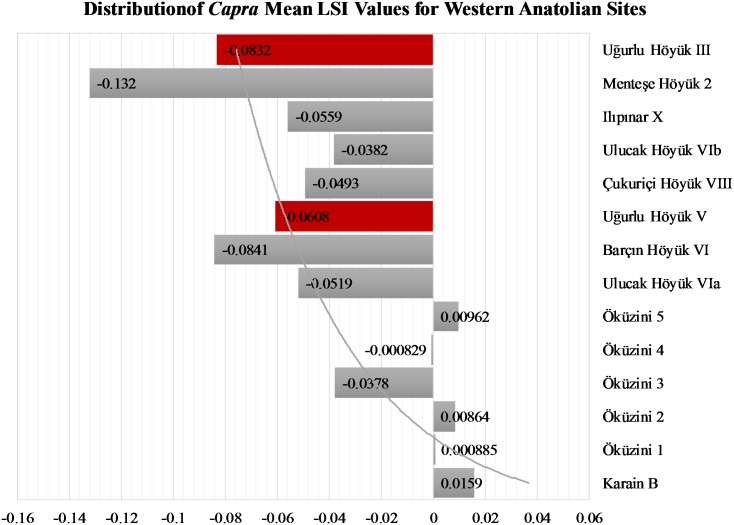
Distribution of *Capra* mean LSI values for western Anatolian sites.

A glance at Fig 10 (see also S7 Table) reveals a similar patterning for goats with slightly greater variation. Similarly, goat populations from Gökçeada fit in the range, overlapping in size with other sub-regions and not representing the smallest size. Thus, it is plausible to assume that Neolithic goats from Gökçeada originated from western Anatolia.

**Fig 11:**
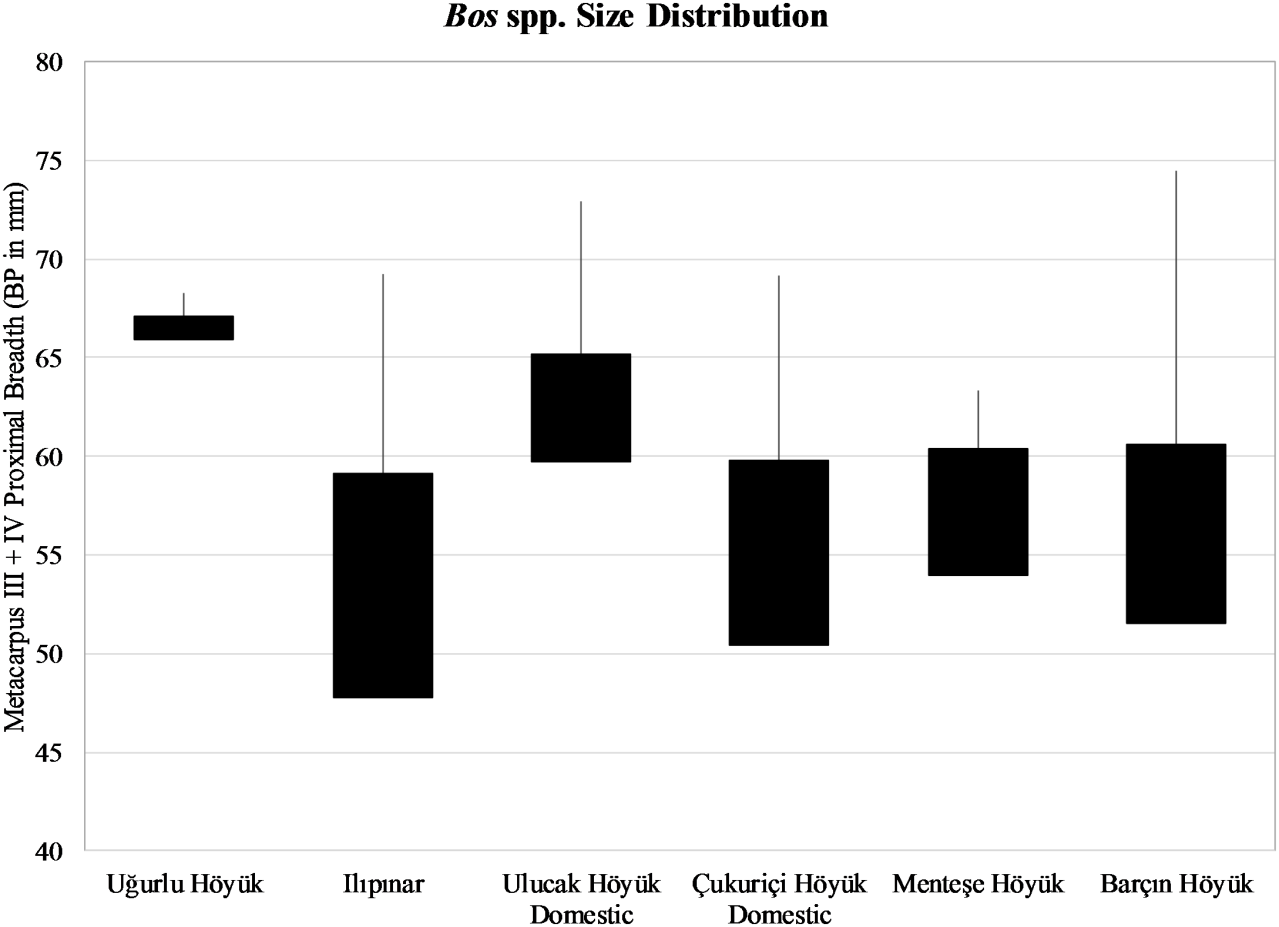
*Bos* spp. size distribution based on the measurement of proximal breadth (BP in mm) in metacarpus III + IV.

For cattle, two proximal metacarpus III + IV breadth measurements, one from stratum V and one from stratum IV, provide us with a glimpse into the *Bos* size range across western Anatolian sites and where Uğurlu Höyük specimens fall within that range. Although neither significant nor conclusive, the two specimens from Uğurlu Höyük are rather large, implying the presence of either large domestic males or aurochs transported from the mainland (Fig 11).

**Fig 12:**
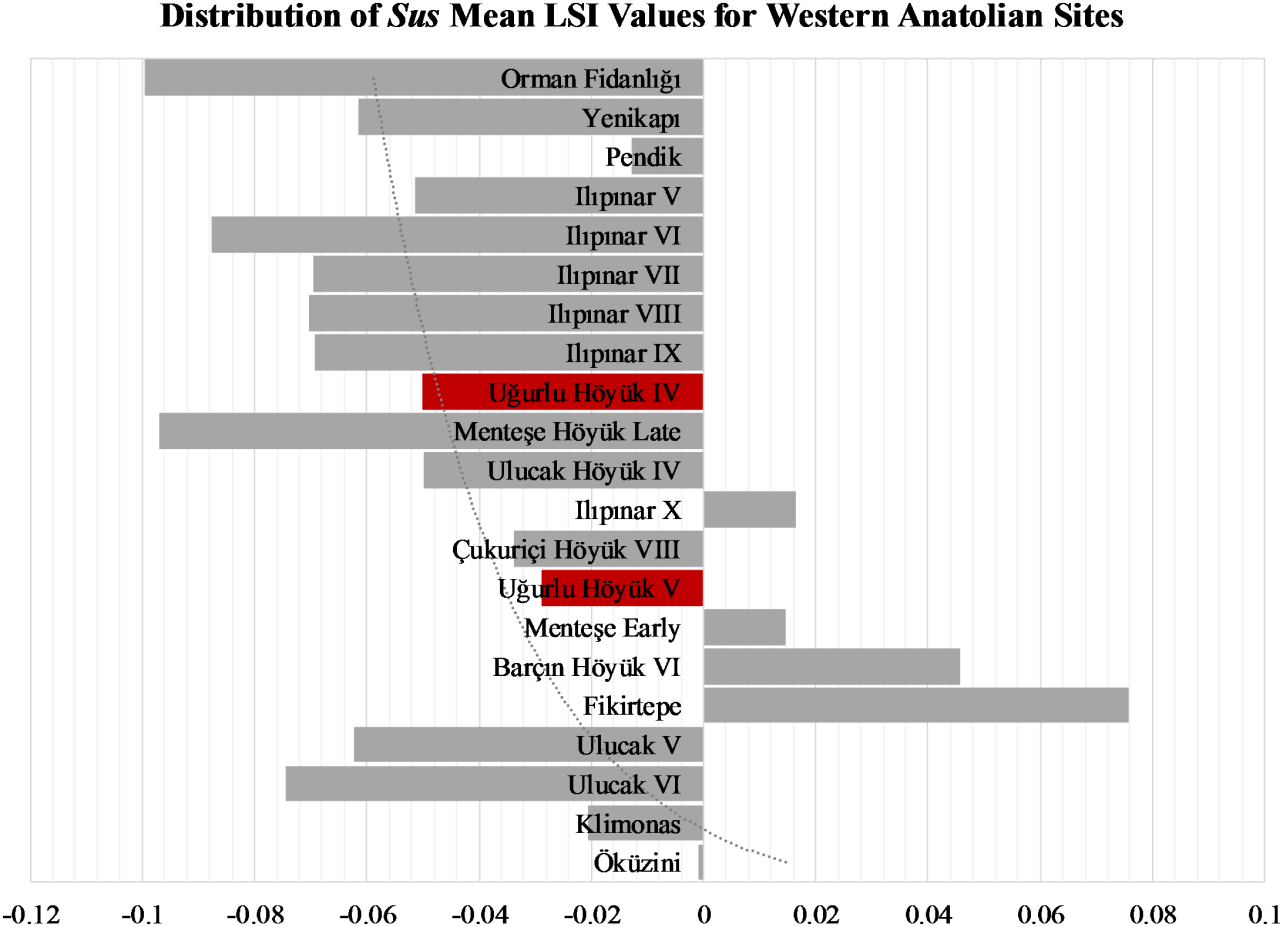
Distribution of *Sus* mean LSI values for western Anatolian sites.

**Fig 13:**
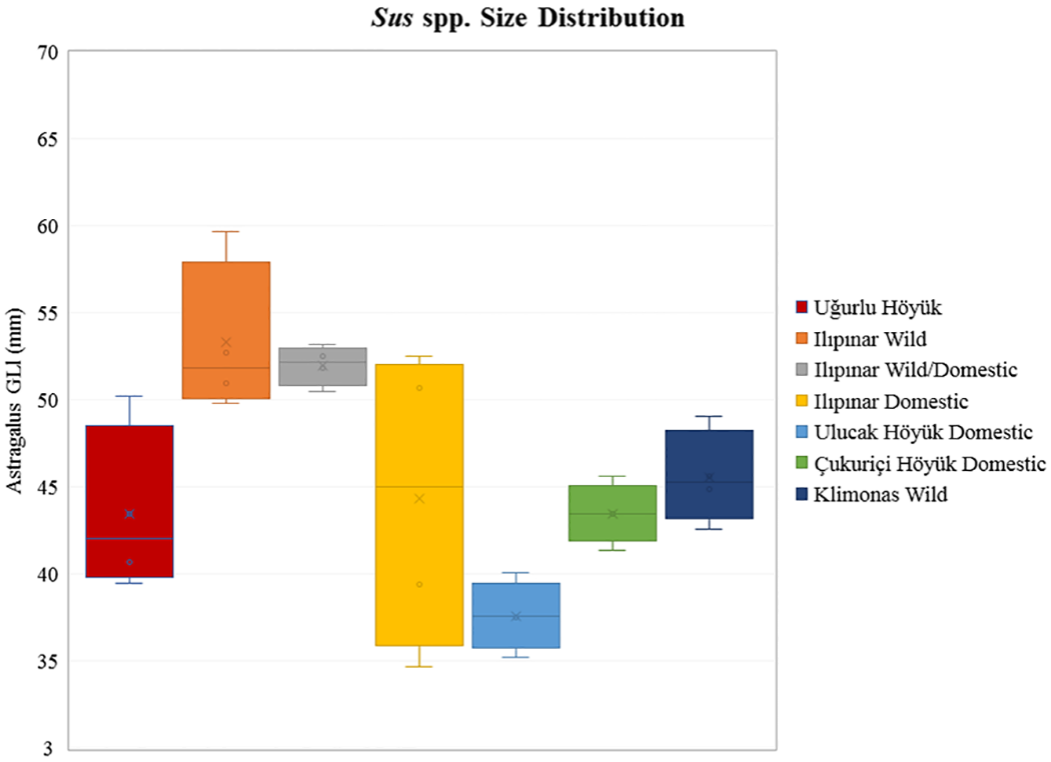
*Sus* spp. size distribution based on the measurement of greatest lateral length (GLl in mm) in astragalus.

The biometric data presented here for Suidae are rather complicated and must be interpreted with caution. On the basis of the mean LSI distributions presented in Fig 12 (see also S8 Table), it is hard to accurately discriminate between wild boars and domestic pigs, since Epipaleolithic Öküzini V and the Cypriot Pre-Pottery Klimonas data attest to the presence of wild boars whose smaller phenotypes overlap with domestic pigs. The amount of variability within and among populations seems pronounced and the degree of overlap between wild boar and domestic pig sizes is large. Based on the LSI patterning, we would postulate that phenotypically wild and large hunted boar populations appear in the assemblages from the Marmara region: at the earliest level of Ilıpınar (X), early level of Menteşe Höyük, Barçın Höyük, and Fikirtepe. In contrast, all the other sub-regions indicate managed domestic pig populations. This patterning, however, would be an artifact of pooling all the measurements from multiple elements to overcome sample size-related biases at the expense of losing resolution. Alternatively, the presence of very large male phenotypes and female-focused hunting strategies may converge to skew the size distribution and make the wild, smaller female individuals fall in the domestic end of the continuum. In this case, a closer look at the osteometric analysis of a single element such as astragalus, which is shown in the box & whisker plot in Fig 13, could be useful. The plot shows suid astragali identified as domestic, wild, and domestic or wild from Ulucak Höyük, Çukuriçi Höyük, Ilıpınar, and Uğurlu Höyük. Data from the Aceramic Neolithic Klimonas from the island of Cyprus [75] are also included to present an island wild boar population as a comparative reference. We must emphasize that the range of size distribution in domestic pigs at Ilıpınar covers domestic pigs from Ulucak Höyük and Çukuriçi Höyük and wild populations from the Cypriot Pre-Pottery Neolithic site of Klimonas and both domestic and wild individuals from Uğurlu Höyük. Therefore, the degree of overlap between the wild and domestic populations presented in the plot confirms that the biometric data are indeed nuanced, calling for careful interpretations.

## Concluding discussion

The zooarchaeological research presented here has addressed the following specific questions to probe animal exploitation strategies at Uğurlu and to add new data to research in the spread of domesticated animals across Neolithic western Anatolia:

*1*. *Did the islanders have a diverse subsistence strategy*, *including foraging and marine resource exploitation*, *or did they heavily rely on livestock management*? *How did the animal economy change through time*?

Although the Neolithic and Chalcolithic inhabitants of Gökçeada exploited a wide range of taxa in varying proportions, remains of three principal food animals—sheep, goats, and cattle—dominate the three Uğurlu Höyük assemblages. Of the taxa, caprines in general and sheep in particular were the primary focus of pastoral economy throughout the cultural sequence. Sheep outnumber goats in all phases although the latter progressively increase and the exploitation of sheep and cattle visibly decline by Chalcolithic.

During the earliest phase of the Neolithic between 7000 and 6500 BC, a more specialized, caprine-dependent animal management regime seems to be represented by both sides of the Aegean Sea; on the mainland Anatolia as documented at Ulucak Höyük VI and Öküzini Cave V.

Between 6500 and 6000 BC, Gökçeada (Uğurlu V) had a three-tiered pastoral economy with a primary focus on caprines and a secondary focus on cattle; pig exploitation was marginal with a proportion around 2%. In contrast, a four-tiered pastoral economy with a primary focus on caprines and secondary, dual focus on cattle and pigs characterizes Çukuriçi VIII and Ulucak Höyük V in the western region. Here, the ratio of pigs increases sharply as a part of progressively increasing taxonomic evenness. A three-tiered animal management system with an equal focus on caprines and cattle, or a shifting primary focus on either caprines or cattle is evident in the Marmara and Turkish Thrace, two sub-regions of northwestern Anatolia, as documented at Fikirtepe, Barçın Höyük VI, Menteşe Höyük early and late levels from the former and Hoca Çeşme from the latter. The suids are represented in marginal proportions in both sub-regions.

Thus, with their three-tiered pastoral economy where hunting boars or raising pigs played a marginal role, northwestern Anatolia and Gökçeada significantly differed from the western region where a four-tiered animal management regime existed. A monotaxic and monodominant taxonomic composition with heavy reliance on cattle may be signaling a more intensified agropastoral economic organization in the Marmara region. It is most likely that a combination of meat, milk, hide, and traction capacity might have made cattle a highly desired commodity supplemented by caprines as a secondary animal source. In this scenario, cattle may have played a vital role in primarily providing secondary products such as dairy, as evidenced by the presence of dairy fats (e.g., [40]), and traction, while caprines may have offered primary products. Given the significant underrepresentation of pigs in the Marmara region, the versatile trio of sheep, goats, and cattle must have facilitated the institutionalization of agropastoral economies across northwestern Anatolia and in transition to Europe. In the western region, in contrast, a departure from caprine-dominated pastoral economy in favor of cattle and, more importantly, of pig, is evident as documented at Ulucak Höyük and Çukuriçi Höyük. With the four principal taxa present, a more diverse and even pastoral production must have emerged with varying emphases on primary and secondary products. In such a pastoral configuration, sheep, goats, and cattle must have been increasingly switched to provide secondary animal products, in addition to their primary products, whereas pigs must have met the demand for meat.

During the latest phase of the Neolithic, between 6000–5500 BC, the species trend in the western region shows a conspicuous continuity with a four-tiered animal husbandry, whereas the sites in the Marmara Region show a greater taxonomic diversity with a sharp drop in cattle and increase in caprine exploitation. The fluctuations in the reconfiguration of taxa in each region and sub-region of western Anatolia mark changing roles of the four vital livestock species through time and across space. This, in turn, may reflect the transformation of Neolithic societies and their agropastoral economies following multiple pathways within a rapidly changing physical and sociopolitical world. As far as the changes identified at Uğurlu Höyük (IV) are concerned, slight but progressive increase in the exploitation of goats and decrease of sheep and cattle most likely reflect the realities of resource management and impacts of environmental circumscription on an island setting. Factors such as mobility, transhumance, and penning, as well as availability, accessibility, predictability, and quality of grazing pastures, water, and fodder must have determined animal management strategies that seem to have varied across taxa. For instance, spatial constraints of islands and resource availability and abundance may pose challenges when herding cattle.

The assemblages representing the early Chalcolithic between ca. 5500–4900 BC come from Ilıpınar VI-IV in the Marmara region and from Uğurlu Höyük III on Gökçeada. At the former, a four-tiered pastoral economy manifests itself in the form of a primary focus on caprines and a secondary focus on cattle and pigs with a sharp increase in cattle and decrease in pigs. In the latter, a three-tiered animal management regime heavily relied on caprines and supplemented cattle as a secondary resource; pigs remained as a tertiary source with marginal proportions under 2%. Hence, it could be argued that a more intensified animal management regime toward a more specialized, caprine-oriented pastoral economy seems to have been established on the island of Gökçeada when taxonomic composition data are combined with mortality and osteometric data.

The lack of or underrepresentation of marine resource exploitation during the Neolithic on the Aegean islands is archaeologically well-documented, as sites from Crete and Cyprus show the same pattern. Munro and Stiner [76] have more recently reported the same pattern during the Neolithic at Franchthi Cave on mainland Greece. In this vein, Uğurlu adds another data point to the same subsistence trend with a focus on livestock, crops, and other terrestrial taxa on the Aegean islands during the Neolithic and early Chalcolithic. As a valid and significant caveat, however, we have to emphasize the critical role of fine-grain approaches to excavation and full recovery of ecofacts, as multiple taphonomic processes and filters might create a scenario of equifinality.

*2*. *How did island habitation affect animal management decisions compared to the mainland Anatolia*? *Did the islanders manage cattle*, *sheep*, *goats*, *and pigs differently*?

The analysis of body part distribution reveals nuanced and complicated data that need to be interpreted cautiously. Due to sample size-related analytical biases, it is not possible to present a diachronic analysis of carcass management for each livestock and game species. Still, with a closer look at the earliest phase of Neolithic, Uğurlu Höyük V, somewhat representative interpretations can be inferred.

For caprines, frequency distribution of body parts suggests that full caprine carcasses were accessed, processed, and consumed on the island. Demography of mortality based on epiphyseal fusion data seems to mimic the ethnographically documented meat-oriented animal management strategy in which caprines are slaughtered between 9 months and 30 months before a potential decline in their meat yield. The dental wear data indicate a progressively younger kill-off pattern from Phase V through III. This, in turn, may be reflecting an increased young male slaughter which is the hallmark of more intensive management and herding of caprines that came to represent specialized pastoral economies of southwest Asia [5]. The fact that mean sheep LSI values for the Marmara and western sites are similar to that of Gökçeada may indicate that the first Neolithic inhabitants of the island may have selected their animals from the same colonizing stock that was dispersing across western Anatolia and into mainland Greece as evidenced by similar LSI values documented at Franchthi Cave [76]. In contrast, caprine populations during the ensuing Chalcolithic phase must have gone through a selective process locally on the island that led to further size reduction to the extent that they sit at the lowest end of the size distribution. Further reduction in caprine body size and progressively increasing young male caprine culling coupled with a more caprine-dominant species trend at Chalcolithic Uğurlu Höyük converge to point to a specialized pastoral economy in which sheep and goats were more intensively managed through time.

Frequency distribution of body parts for cattle indicates that complete carcasses were locally processed, consumed, and discarded. Combined with evidence for a decline in cattle proportions through time and a shift in demographic mortality toward older animals allude to a pastoral economy where cattle might have been kept for their capacity for traction and/or dairy production. The extremely small sample size (N = 2) for osteometric data does not permit interpretations going beyond predictive assertions. But, based on the combined species trends, age, size, and body part data, we lean toward the idea of small-scale cattle herding that was a part of the pastoral economy on the island during the earlier stage of the Neolithic dating to around 6500–6000 BC. Accordingly, transportation of selected aurochs cuts hunted on the mainland or the presence of wild stock on the island does not fit the data we have from Uğurlu Höyük.

A trend of progressively declining cattle proportions through the late Neolithic and early Chalcolithic on Gökçeada merits further examination. Although we do not have data to directly test whether cattle persisted on Gökçeada beyond the early Chalcolithic, zooarchaeological insights from Neolithic Cyprus, where cattle disappeared quickly after their initial introduction to the island during the Pre-Pottery Neolithic and until the Bronze Age, offer hints [77]. It is known that cattle require different herding strategies than caprines or pigs and may be incompatible with an island setting because of the realities of resource management and impacts of environmental circumscription. Factors such as mobility, transhumance, penning, as well as availability, accessibility, and predictability of high-quality grazing pastures, water, and fodder must have impacted the degree and scale of cattle herding on Neolithic Gökçeada where the large-sized cattle could have quickly depleted forage. On the contrary, we must emphasize that numbers of cattle and sheep do not necessarily reflect their true economic value or caloric contribution to ancient diets as per significant size/weight difference between these taxa; cattle will provide more meat per carcass than caprines or pigs. As such, instead of NISP, MNE or MNI values *per se*, bone-weight data must also be factored in to interpret species trends. At Uğurlu Höyük, bone weight data, too, confirm cattle’s progressively declining role on the island (see S3 Table).

For the game taxa such as wild boars, the body part profile data indicate a forelimb- and hind limb-dominated frequency distribution in the Neolithic. The preponderance of nutrient-dense and high economic utility anatomical parts raises the question of whether the game animals were introduced onto the island from the mainland by the first settlers and then sustained viable, natural populations through the processes of feralization and hybridization. Alternatively, it could be argued that the multidirectional and recurrent interactions and voyages between the mainland Anatolia and the island of Gökçeada may have resulted in trading animals/animal products for material goods and perhaps occasional transportation of selected game cuts to the island. Stating the obvious, testing and positively verifying this proposition requires evidence beyond body part distribution data. Toward this end, the osteometric analysis of archaeofaunal specimens and size comparison among the mainland and island Neolithic sites may shed light onto this specific question, as well as stable isotope analysis of these remains [42]. This latter scenario would be legitimate and plausible based on the archaeologically documented fact that domesticated animals went through a gradual size decrease following their initial management beginning around 10th millennium BC in southwest Asia ([6] and references therein) and references therein). Furthermore, the fact that island faunas go through rapid size reduction due to genetic bottle-necks created by island insularity phenomenon (sensu [78]) represents an opportunity to test whether Gökçeada assemblages show the effects of this phenomenon, or instead, they display affinities with the mainland as a result of the colonization. It is of particular interest to test whether the pace and magnitude of size decrease increased or decreased after the initial Neolithic colonization.

Based on the archaeologically documented material exchanges between early farming populations, it is plausible to hypothesize a process in which animals and their parts and products were traded for goods among early farmers across western Anatolia. To further complicate the matter, as archaeologically documented for pigs, hundreds of years of introgression between feralized domestic stock and wild herds would manifest itself in the form of variable mix of traits and sizes ([79]: 836). This, in turn, further exacerbates the situation, since a mixture of wild and domestic genetic and morphological characteristics would be osteologically reflected in the zooarchaeological record. As Albarella, Dobney, and Rowley-Conwy [80] have documented, using biometry alone to accurately discriminate between wild and domestic forms will not generate comparable and consistent results due to population-specific intra-species size variation (see also [79]: 837). Albarella and colleagues [81] note that in the islands of Corsica and Sardinia wild, feral, free-range and fully domestic pigs interbreed regularly and thus create a biological continuum that could not possibly be identified morphologically or biometrically, but behaviorally. As such, they treat all specimens from the family Suidae as a single biological entity without attempting to assign them “wild” or “domestic” status ([81]: 292). In addition, application of multiple exploitation strategies, hunting, and seasonal mobility and transhumance, may lead to distorted biometric and demographic patterning that further complicates our understanding of Neolithic animal management systems and obscure zooarchaeological signatures [30].

The clarification of the family Suidae’s status on the island of Gökçeada and particularly the verification of the presence of domestic pigs may potentially shed new light on the timing and directionality of the dispersing farming populations. All four livestock species, including domestic pigs with distinctively small phenotypes, are documented in the Aegean region at Ulucak VI during the early seventh millennium BC, alluding to a rapid westward movement of domestic animals across southern Turkey following a coastal route by sea or land [6].

The fact that the earliest Neolithic communities in the Marmara region relied heavily on caprines and cattle but not pigs affirms that the colonization process that delivered crops and animals, along with all the other socioeconomic and ideological elements, was rather complex. The magnitude of difference in *Sus* mean LSI values from Menteşe Höyük early to late around 6000 BC and from Ilıpınar X to IX around 5800 BC onward marks a shift from hunting wild boars to raising domestic pigs in the Marmara sub-region of northwestern Anatolia. Arguably, this would be deemed as a material reflection of the rapidly evolving sociocultural landscape across western Anatolia. In this vein, the Aegean region may have sent the waves that added domestic pigs to the evolving agropastoral lifeways in northwest Anatolia, albeit not in a unidirectional fashion.

Arbuckle and colleagues ([6]:8) further argue for the presence of two distinct colonization pathways corresponding with distinctive animal economies and ceramic technology: 1) caprines, cattle, and pigs and the initial Aceramic expansion of Neolithic lifeways and with later Red Slipped Burnished Ware horizon during the late eight-seventh millennium BC into coastal and inland SW and western Turkey; and 2) domestic caprines and cattle associated with Dark Faced Burnished Ware tradition from the interior Anatolian Plateau. Thus, would the presence of domestic pigs alone place Uğurlu Höyük within the first colonization pathway and directly link it to southwest and western Anatolian domain? Or would the absence of domestic pigs suffice to establish spatiotemporal relationships between the Marmara and Thrace regions and Gökçeada? The answers to these questions are nuanced and would have to incorporate more than presence or absence of taxa and/or ceramic techno-typology.

Subsequently, although it is highly likely that the remains of both domestic pigs and wild boars are present in the assemblages from Uğurlu Höyük, we don’t intend to rush to a conclusion without incorporating other lines of evidence such as ancient DNA and stable isotopes analyses to clarify the status of the family Suidae on the island of Gökçeada. It seems that selective processes continued to operate, although at a slower rate, to lead to further size decrease in Neolithic pigs at Uğurlu Höyük, as well as occasional hunting of boars.

Neolithic Uğurlu is characterized by rich material remains as well as striking evidence for early craft specialization and long-distance communications. General parallels to Uğurlu pottery have been found at western Anatolian sites. Several forms of pottery from Phase V, the oldest Pottery Neolithic phase at Uğurlu, show parallels with Hoca Çeşme in Turkish Thrace as well as Aktopraklık, and the basal layers of Menteşe in the Marmara region. But when incorporating other lines of evidence such as lithic analysis, the story becomes more intricate, revealing spatiotemporal relationships beyond Anatolia

Accordingly, based on ceramics and techno-typological and source analyses of lithics from Uğurlu Höyük, relationships between the island of Gökçeada and central Anatolia, Marmara, Thrace, the Balkans, and other Aegean islands can be established. As such, with or without domestic pigs, Uğurlu Höyük on the island of Gökçeada in the northeastern Aegean Sea compellingly demonstrates that Neolithic dispersal was a polynucleated and multidirectional phenomenon and that its spread was not a unison and unidirectional phenomenon sweeping across the land, replacing everything on its way, and delivering the same Neolithic package everywhere. This complex process most likely generated a diversity of human-animal interactions. Environmental matrices, behavioral ecologies of involved taxa, and human agency must have shaped fluid and versatile interactions as evidenced in zooarchaeological assemblages from across the region. Attempting to develop a template that could characterize a typical Neolithic animal management system is not only impossible, but also unnecessary and unproductive, as it would prevent us from identifying peculiarities and idiosyncrasies that existed in past human-animal relationships.

Domestication of animals is a complex phenomenon that involves a continuum between resource management, domestication or morphological changes associated with management, and fully-developed animal husbandry or intentional and intensive human management of animals (e.g., [5, 31]). The study of this phenomenon, in turn, requires approaches beyond binary status assignment and using single lines of evidence and/or monocausal explanatory frameworks. It is difficult to clearly establish domestic status when a full suite of morphological and genetic characteristics is unavailable. In the same vein, studying the dispersal of early farmers from southwest Asia into southeast Europe via Anatolia requires a rigorous methodological approach to develop a fine-resolution picture of the variability seen in human adaptations and dispersals within complex and rapidly changing environmental and cultural settings. For this, the whole spectrum of human-animal interactions must be fully documented for each sub-region of southwest Asia and circum-Mediterranean. Building upon and adding to the high-resolution regional-scale project spearheaded by Arbuckle and colleagues [6] to document the westward spread of domestic animals across Neolithic Turkey, Uğurlu Höyük on the island of Gökçeada in the northeastern corner of the Aegean Sea, an area previously underinvestigated and neglected, offers us an additional piece of evidence and new data elaborating the nature of the Neolithic dispersals.

The results of zooarchaeological research presented here align well with the findings of Arbuckle and Atici [30] and Arbuckle and colleagues [6] in that the initial diversity in animal management systems of the Pleistocene-Holocene transition in southwest Asia continued deep into the Neolithic and Chalcolithic with the dispersal of fully developed agropastoral lifeways of early farming populations into southeast Europe. The first settlers of Gökçeada were agriculturalists and they introduced domestic sheep, goats, cattle and pigs to the island as early as 6500 years BC. The early Neolithic has signs of continuity, but the cultures of island and mainland clearly diverge. Differences in material culture may be deliberate expressions of local identities within a wider cultural setting.

## Supporting information

S1 Table Radiocarbon dates from Uğurlu Höyük with lab references numbers, sample numbers, materials dated, and BC calibration limits for one standard error. (XLSX)

S2 Table General characteristics detailing the taphonomic histories of the three Uğurlu Höyük assemblages. (XLSX)

S3 Table Taxonomic composition in the three Uğurlu Höyük assemblages using Number of Fragments, Minimum Number of Elements, and Bone Weight in grams. (XLSX)

S4 Table List of sites used in this paper with data including region, phase, chronology, author, and relative abundance of *Ovis*, *Capra*, *Bos* and *Sus* based on %NISP (After Arbuckle et al. 2014). (XLSX)

S5 Table Frequency distributions of body parts based on %MNE counts in main taxa. (XLSX)

S6 Table Mean LSI values and standard deviations for sheep from late Pleistocene-early Holocene sites in western Anatolia. Author information and links to online databases are also included.
(XLSX)

S7 Table Mean LSI values and standard deviations for goats from late Pleistocene-early Holocene sites in western Anatolia. Links to online databases are also included.
(XLSX)

S8 Table Mean LSI values and standard deviations for *Sus* from late Pleistocene-early Holocene sites in western Anatolia. Author information are also included.
(XLSX)
